# The Foreign Journals: Pathology, Practical Medicine, and Therapeutics

**Published:** 1841-10

**Authors:** 


### PATHOLOGY, PRACTICAL MEDICINE, AND THERAPEUTICS.

*Cases illustrating the Pathology of the Spinal Marrow.* By M. PRUS, Physician to the Salpêtrière.

THIS paper is an account of a number of cases highly important in illustration of a class of diseases hitherto very imperfectly understood. We can merely give the chief features of each case, referring our readers for details to the original paper.

CASE I. Contraction of the extensors and flexors of both inferior extremities; preservation, but alteration of the sensibility of these parts. Progressive paralysis of the sphincters of the rectum and bladder. Death. Gelatiniform softening of the two inferior thirds of the spinal marrow.

CASE II. Sudden loss of speech; deviation of the mouth to the right; progressive diminution in the voluntary power of the left arm; incomplete paralysis of motion in the left side of the face without any alteration of sensibility; involuntary discharge of saliva; dyspnœa carried to commencing asphyxia; death; superficial softening of the whole circumference of the medulla oblongata.

Case iii. Pricking and loss of sensibility of all the limbs, especially of the inferior; incontinence of urine; sloughs on the sacrum; death; slight softening of the posterior columns of the spinal marrow in the portion constituting the lumbar swelling.

Case iv. Loss of motion in the inferior extremities; sensation preserved; acute pains continued during many years in the lateral parts of the chest; purulent expectoration; hectic fever; softening of one portion of the spinal marrow; spinal meningitis; caries of the seventh, eighth, and ninth dorsal vertebræ; abscess communicating with the bronchiæ by means of numerous fistulæ; compression of the spinal nerves at their passage through the foramina of the carious vertebræ.

Case v. Progressive paralysis of motion only in all the limbs; paralysis of the sphincters of rectum and bladder; spasmodic respiration; sudden death in going to stool; double softening of the spinal marrow, that is, in the cervical and lumbar portions; the gray matter of the cord invisible in the diseased parts, almost so in others.

Case vi. Paralysis of motion only in the four limbs, which are slightly contracted; progressive paralysis of the sphincters of rectum and bladder; reddish softening in the middle lobe of right hemisphere of cerebrum, and in anterior lobe of left hemisphere; spinal marrow perfectly healthy.

Case vii. Paraplegia during three years; exostosis of the left clavicle; treatment by mercury and sudorifics; successive disappearance of the exostosis of the clavicle and of the paraplegia. *Revue Médicale. Décembre,* 1840.

---

### *On the Puerperal Fever observed in the Hôtel Dieu at Paris in* 1840. By M. Bourdon.

The principal interest of this paper is in the account of the morbid appearances. In one case the only appreciable lesion was a slight effusion of reddish serum into the peritoneal cavity, holding in suspension small flocculi. In another there were all the anatomical alterations which characterize uterine phlebitis, but the most minute examination failed to discover the slightest quantity of pus deposited in the serous or synovial cavities, or infiltrated into the cellular tissue of muscles or of any organ. In a third case there were all the degrees of softening of the uterus described as gangrenous metritis, or putrescence of the uterus. Besides this gangrenous softening of the internal surface of the uterus, a perforation through the whole thickness of its walls had taken place. There were also traces of severe peritonitis, with fetid blackish effusion, the serous cavity communicating externally by perforations of the uterus. The subperitoneal cellular tissue and the subjacent muscles were infiltrated with pus of the same nature. Most of the organs were softened, and the small intestines were ulcerated without traces of surrounding inflammation. Another case offered all the characters of the pyrogenic fever of M. Voillemier. Pus was infiltrated in the cellular tissue of the limbs at their posterior part, and under the peritoneal covering of the uterus, without trace of phlebitis, inflammation of the uterine lymphatics, or metastatic abscess in the parenchymatous organs. In a fifth patient all these lesions coexisted, pus being united in a focus, or diffused in the subperitoneal cellular tissue of the pelvis, almost all the organs being softened, but especially the right lung, which broke up or tore like the tissue of the liver, without losing its property of floating in water.

Some alterations were constant in all the autopsies; a detritus, blackish or of the colour of wine lees, of a sickly odour, forming a layer of variable thickness over the internal surface of the uterus, the quantity being sometimes so great as to cause considerable enlargement of the uterus. It may be considered as the coagulated product of the lochial secretion more or less putrefied, as the layer of false membrane which sometimes covers the internal surface of the uterus may be regarded as a peculiar exudation, independent of all inflammatory action. The blood was remarkably fluid in the heart and vessels, and in every case there was softening, more or less marked, of the parenchymatous organs and of the heart.

The only means which appeared useful in the treatment were methodical compression of the abdomen, and ipecacuanha. The author thinks that the latter does good, not only by exciting vomiting, but that it also acts in a "special unknown manner." Camphor in large doses, mercurial embrocations, blood-letting, and other usual means, were tried without good effect. Injections into the uterus appeared to be of service by removing the collections of fetid detritus.

*Revue Médicale. Juin*, 1841.

During the last few weeks puerperal fever has been epidemic in Paris; and though at first of slight severity, it has become rapidly so intense that M. Dubois has been obliged to clear his wards.

*Bulletin Général de Thérapeutique.* 15 *et* 30 *Juillet*, 1841.

---

*On the Contagiousness of the Pseudo-membranous Inflammation of the Mouth, Pharynx, Œsophagus, and Stomach.* By Dr. KESSLER, of Samter.

ON the 1st of December, 1840, a robust woman, twenty years old, after exposure to cold, was seized with the usual signs of this disease, which continued till the thirteenth day, when the false membranes began to be thrown off, and the whole condition of the patient improved. After some days more she completely recovered.

Up to the fifth day of her illness she was nursed and watched by one of her relations, a woman named F.; she however became ill, and then a woman forty years old, and of rather a weak constitution, was attacked with the same disease, with a remarkable predominance of the nervous symptoms. The place of both these was then taken by an unmarried woman, S., twenty-seven years old, who had been reduced by a very laborious life, and she also fell ill with the same symptoms on the 9th of December.

At the same time another woman, G., who lived in the neighbourhood, and had often waited on the preceding, sickened with the same disease. In her it assumed many of the characters of typhus abdominalis, and she died on the 20th of December.

Another woman, twenty-eight years old, of a rather weak habit, and very excitable, now came to attend on F. and S. and waited on them. She became ill on the 15th of December with the same symptoms, and the disease in her took the same course as in them, the nervous symptoms being more than commonly predominant.

Her husband, a man fifty years old, who had drunk hard, and now attended steadily on his sick wife, fell ill with nearly the same symptoms on the 20th of December, and in him the disease deviated from its usual course into an attack of delirium tremens.

There were thus six cases of this membranous angina which in their mode of spreading evinced a contagious nature, or which, at least, seemed to spread only by contagion The patients were of different ages and constitutions, occupied different positions, led different kinds of life, and, for the most part, inhabited different houses.

*Medicinische Zeitung. Mai* 19, 1841.

*Ileus from Hypertrophy of the Pancreas.* By Dr. Holscher, of Hanover.

The subject of this case was a strong and apparently robust man, forty-eight years old, in easy circumstances, and the father of a numerous but scrofulous family. He had occasionally lived freely; but during the last six or eight months of his life, whenever he made a full meal, he always felt a great oppression at the stomach and umbilicus, and was obliged to drink a large quantity of water, as much sometimes as two or three quarts. His bowels were very costive, but still he scarcely thought himself unwell till the 20th of July, when he stated that, four days previously, after having eaten and drunk a good deal at a friend's house, he thought he had caught cold. A surgeon had given him an emetic on the following morning, and he had vomited; but since that time, though he had taken various purgatives, his bowels had never been open. He suffered from a constant desire to discharge fæces, and was always thirsty, but as often as he filled his stomach he vomited its contents again. He had but little pain but his anxiety was inexpressible. Pressure on the precordia gave no pain, and there was no trace of inflammation, but the abdomen felt rather tense. He was in a cold sweat and his pulse was 130; he had hiccup, and frequently vomited a clear, watery, acid-smelling, almost colourless fluid. In spite of a variety of means the patient died on the 22d.

At an inspection thirty-six hours after death, the abdomen exhibited neither effusion nor any trace of inflammation. But it was found that the pancreas had grown to the size of the head of a four-months' fœtus, and had so enclosed the duodenum for about three inches of its length, that its canal would not permit a goose-quill to pass. Anterior to this constriction the duodenum from the pylorus onwards was so distended as to form a kind of second stomach, in which for a long time past the food must have always undergone a considerable dilution before it could pass through the stricture. The hypertrophied pancreas had lost its normal granulated character; it had become softer, more succulent, and more fleshy, but when cut through it exhibited neither tuberculous matter nor any appearance like cancer or fungus hæmatodes.

*Hannoversche Annalen.* 1840. Heft ii.

---

*Intestinal Strangulation produced by the Peduncle of a Fatty Tumour developed in the Cellular Tissue of the Mesentery of a Mare.* By M. Leblanc.

This mare died after having suffered for fifteen hours with violent colic; and at a short distance from the stomach the small intestine was strangulated by the elongated peduncle of a fatty tumour developed in the cellular tissue of the mesentery. The peduncle completely surrounded the intestine, arising on the left side of the mesentery, and being again attached by the fatty tumour which was appended to it.

*Bulletin de l'Académie Royale de Médecine. Mars* 31, 1841.

---

*On Hemeralopia which was Epidemic in the Department Bouches-du-Rhône.* By Dr. Frechier.

Epidemic hemeralopia has often been observed. In the present instance it was first observed at Mausanne in the commencement of March last, affecting pregnant women especially, but sparing neither age, sex, nor temperament. In some it simply enfeebled vision after sunset; others were completely blind at night, although their vision was perfect during the day; and in others vision was imperfect even at mid-day, although it was not entirely abolished at night. The duration of the affection was about seven or eight days; the constituent parts of the eye were unaltered; indeed, except in the cases in which pregnancy was concomitant, the affection was absolutely isolated. The cause was evidently general and diffused, but its nature is subject for conjecture.

*Bulletin Général de Thérapeutique.* 15 *et* 30 *Avril,* 1841.

*On the Composition of the Urine in Pregnancy and under Disease.* By M. Donné.

At the sitting of the Royal Academy of Sciences, May 24, 1841, M. Donné presented a memoir on the above subject. The urine during pregnancy contains less uric acid and phosphate of lime than in the natural state, a difference easily comprehended on considering the elements necessary for the formation of the bones of the fœtus. The crystallization of the salts of the urine is thus modified in a manner so remarkable, that by simple inspection, without examining the females, M. Donné has in more than thirty cases recognized the state of pregnancy at its different periods.

In a healthy man the urine contains iron; in chlorosis it cannot be detected by the ordinary reagents, but it is discovered after the administration of ferruginous preparations, and thus the examination of the urine affords valuable indications as to the necessity for these medicines. Thus in certain forms of chlorosis, the urine contains iron, and in such cases iron will not prove serviceable. We may know also that the cure of chlorosis is complete if the urine contain iron several days after its internal administration has been suspended.

In typhoid fever the crystals of the urine present a radiant pearly aspect, and assume the appearance of crystals of phosphate of ammonia; but this character is not very important, for it is met with in other diseases, as in pneumonia.

M. Donné also announced that he had injected milk into the serous cavities of dogs, and even into the veins, and he is convinced that when the milk is pure, it produces no bad effect, but circulates in the vessels as the blood.

*Gazette Médicale de Paris.* 29 *Mai*, 1841.

---

*On the Use of Oxalic Acid.* By Dr. Nardo.

Dr. Nardo has employed this acid in inflammations of mucous membranes, and finds that its antiphlogistic action is more marked than that of any other vegetable acid, possessing the property of instantly calming the severe pains which frequently accompany inflammation of the mucous tissue. He has used it with success in acute and chronic affections confounded under the name of angina, in different inflammations of the mouth, aphtha of newborn infants, gastritis, and gastro-enteritis.

The following is the formula he prefers:

| | |
|---|---|
| Solution of gum arabic . | 3 ounces, (94 gram.) |
| Oxalic acid . . . | 1 to 2 gr. (15 to 30 cent.) |
| Gooseberry syrup . . | 1 ounce, (32 gram.) |

A tablespoonful (cuillerée à bouche) to be taken slowly at short intervals.

*Journal des Connaissances Médico-Chirurgicales. Juin* 1841.
(From *Observatore Medico.*)

---

*On the Treatment of Scrofulous Affections by the preparations of Walnut Leaves.* By G. Negrier, Professor at the Preparatory School of Medicine at Angers.

M. Negrier has used the above remedy in seventeen scrofulous children, nine of whom had osseous enlargements with caries; seven ulcerated glands; one several swollen cervical glands with scrofulous ophthalmia of both eyes. Each patient took daily two or three cups of infusion of bruised walnut leaves sweetened with sugar or honey, and a four-grain pill of the extract of the leaves, or a spoonful of a syrup prepared with eight grains of the same extract to ten drachms of syrup. All the sores were washed with a strong decoction of the leaves, and covered with linen compresses steeped in this decoction, or poultices made with flour and the decoction. Seven of the patients were completely cured after six months of this treatment, and five nearly so; and M. Negrier concludes that the walnut leaves are superior to all other antiscrofulous remedies.

*Bulletin Général de Thérapeutique.* 15 *et* 30 *Mai*, 1841.

*Intussusception and Separation of a part of the Ileum.* By Dr. Forcke, of Goslar.

A mechanic, forty-nine years old, had been in the campaigns of 1812-13-15, and from that time had very often suffered from colic and other signs of abdominal disorder. In March, 1838, when first seen, he had not left his bed for five months; he was excessively emaciated, pale, and broken down by irritative fever; during all this time also he had suffered from costiveness, gripes, and vomiting. On examining the abdomen a large, long, hard, and sensitive tumour was felt on the right side, which occupied the usual position of the cæcum and ascending colon. Various antispasmodic means produced but little effect on the symptoms from which he suffered. The only ease he received was derived from his bowels being opened, and from the use of enemata containing opium. The pains in the tumour and in the abdomen rising to a fearful height, the patient was ordered calomel and opium, and emollient poultices over the whole abdomen. These diminished the pain, and produced free evacuations, rest, and sleep, but the tumour remained unaltered. On the 7th of April a severe hemorrhage from the intestines occurred, and was followed by the discharge of pus, which however ceased when the patient took large quantities of lime water.

On the 28th of April the patient had an enormous discharge from the intestines, which made him feel as if something had been torn out of his abdomen, and induced him to examine the evacuation. He found therein in the midst of fæces and blood, a portion of ileum which measured two feet nine inches and a half along its convex border; it was dark and livid, but had a firm texture, and was still connected with a portion of mesentery. Its discharge was followed by a slight hemorrhage and a considerable secretion of pus, which however again ceased after using lime water. The patient was almost speechless with exhaustion, but the pain and the tumour had nearly disappeared. Tonics were administered, the fever diminished, and the strength rapidly increased. In the following October he had regained all his former strength and was hale and hearty, except that, on severe bodily exertion, the sensation of tension around the umbilicus, and of dragging of the stomach, would still return.

*Hannoversche Annalen.* Bd. iii. Hft. iv.

---

*Case of Deficiency of the Uterus.* By Dr. Wehr, of Kassel.

The patient was a little woman, who was born of weakly parents, and died aged fifty-four. At the age of ordinary puberty her breasts did not enlarge, nor did she either then or ever after menstruate, though at almost regular monthly periods she had pains in the limbs and other parts of the body. Throughout life she enjoyed moderately good health, and suffered from no singular symptoms; she died of pneumonia.

After death the clitoris was found very much developed, but there were scarcely any traces of nymphæ, and but little hair about the external organs. The vagina was very small, the hymen perfect, the orifice of the urethra large. The form of the uterus was preserved in the arrangement of the peritoneum, but very little of its substance was found in what corresponded to its body and cornua, and only a little more in its cervix. In the left broad ligament was a small tubercle. The left ovary resembled only an elongated flattened transparent cyst full of fluid; the right ovary was in nearly the same state, but appeared connected by a little tubercle with the rim of the pelvis. The Fallopian tubes were of about the normal length, but their size was small and proportioned to that of the uterus and ovaries, their abdominal orifices were small and surrounded by a few imperfect fimbriæ. The vagina was small; its walls were thin and but slightly wrinkled internally. The orifice of the uterus was flaccid; the cervical part of the cavity was wide, and was continued through the body into two separate cornua.

*Casper's Wochenschrift. Mai* 8, 1841.

*On Syphilis in Pregnant and recently-delivered Females.* By M. HUGUIER.

M. HUGUIER has made a long series of observations on this subject, and has arrived at the following conclusions:

1. The sole primitive phenomenon of syphilis is sometimes a pustular eruption at the vulva. This is often the commencement of chancre. 2. Purgatives are not proper for pregnant females tainted by syphilis. 3. Abortion in syphilitic females is rather in consequence of the exhibition of mercury than of the disease itself. 4. The venereal disease has not the grievous influence on pregnant females which is attributed to it. In twenty-seven females in this condition, only three are dead, and probably their death resulted from lesions unconnected with syphilis. 5. The mercurial treatment does not always preserve the infant nor protect from relapses, and often produces serious results. 6. The sublimate is the most dangerous among the mercurials, and mercurial frictions are preferable to it. 7. Syphilis is very often hereditary; and it ordinarily declares itself from three to thirty days after birth. This last remark is important, for if the child be examined on the first or second week, it is declared healthy and sent out to nurse, probably with the effect of infecting the female.

*Archives Générales de Médecine. Aout*, 1840.

